# Sewill’s Dental Caries

**Published:** 1885-05

**Authors:** 


### SEWILL'S DENTAL CARIES.

Dr. Searle, in the April number of *Archives*, reviews Sewill's "Dental Caries" in an able manner, but the game seems scarcely worth the candle. Mr. Sewill is a rhetorician, who, if we mistake not, obtains his facts at second-hand. We do not understand that he is an original investigator, one who is qualified to speak from his own knowledge. When such an one as Charles Tomes makes the assertions in which Sewill's work abounds, it will be time for those possessed of an exact knowledge to speak at length.

